# Perfect Complementarity Mechanism for Aphid Control: Oligonucleotide Insecticide Macsan-11 Selectively Causes High Mortality Rate for *Macrosiphoniella sanborni* Gillette

**DOI:** 10.3390/ijms241411690

**Published:** 2023-07-20

**Authors:** Yelizaveta V. Puzanova, Ilya A. Novikov, Anastasiya I. Bilyk, Alexander K. Sharmagiy, Yuri V. Plugatar, Volodymyr V. Oberemok

**Affiliations:** 1 Department of Molecular Genetics and Biotechnologies, V.I. Vernadsky Crimean Federal University, 295007 Simferopol, Crimea; 17obruchka@mail.ru (Y.V.P.); i.nowikow2012@mail.ru (I.A.N.); bilyk.ai97@mail.ru (A.I.B.); 2 Nikita Botanical Garden, National Scientific Centre, Russian Academy of Sciences, 298648 Yalta, Crimea

**Keywords:** oligonucleotide insecticides, chrysanthemum aphid, plant protection, antisense technologies, gene expression, nuclease activity, eco-friendly insecticides

## Abstract

*Macrosiphoniella sanborni* is a widespread pest of *Chrysanthemum morifolium* that causes significant damage to world floriculture. Chemical insecticides and biological methods of control have a number of disadvantages that can be improved by using oligonucleotide insecticides. In this article, we present, for the first time, the results of using oligonucleotide insecticides, for which the target sequence is an internal transcribed spacer (ITS) in a polycistronic rRNA transcript. The mortality of wingless aphid individuals after a Macsan-11 treatment was recorded at a level of 67.15 ± 3.32% 7 days after a single treatment with a solution at a concentration of 100 ng/μL and 97.38 ± 2.49% 7 days after a double treatment with a solution of the same concentration and a daily interval. The contact use of the control oligonucleotide (ACTG)_2_ACT-11. as well as the oligonucleotide insecticides Macsan-11(3′) and Macsan-11(5′) was not accompanied by insect mortality. Given the high variability in the internal transcribed spacer, which has proven to be a promising target for the action of oligonucleotide insecticides, it is possible to create selective preparations. This study showed the prospects of ribosomal insect pest genes as targets for the action of olinscides, and also demonstrated the high specificity of such insecticidal agents.

## 1. Introduction

The first reports on the successful use of DNA insecticides (oligonucleotide insecticides, or olinscides) for the control of insects in the suborder *Sternorrhyncha* (order *Hemiptera*) date back to 2020 [1] and indicate a high degree of vulnerability of this group to the effects of unmodified antisense oligonucleotides. When starting our experiments on aphids, we took into account that the high reproductive potential of the pest can become an obstacle to the rapid achievement of a substantial lethal effect. However, because they have thin exoskeletons that are not protected by a powerful wax coating [2], aphids are a convenient model organism for the testing and development of oligonucleotide insecticides.

**Figure 1:**
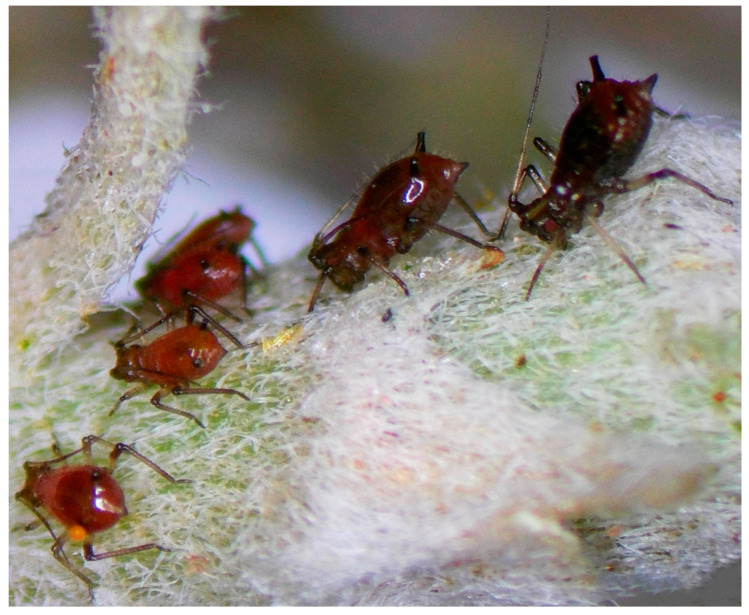
Aphid-affected Chrysanthemum morifolium.

The distribution of the chrysanthemum aphid is associated with greenhouses, horticultural centers, and small private farms around the world (Figure 1). *M. sanborni* is dangerous not only for the direct damage it causes to buds and young shoots due to feeding on phloem juice, but also for its ability to spread a number of RNA viruses [2], including chrysanthemum virus B, a chrysanthemum vein mottle virus strain, potato virus Y, turnip mosaic virus, watermelon mosaic virus 2 [3], and Moroccan watermelon mosaic virus (MWMV) [4]. In addition, aphids provoke the development of sooty fungi [5]. All these factors deplete the plant, reduce the efficiency of photosynthesis, and disrupt metabolism, affecting the profitability of flower production [6] and subsequently leading to economic loss [7]. 

The wave of “green” technologies and the need to take care of the environment have contributed to increasing consumer demands for manufactured products [8]. To date, chemical insecticides [9,10] and biological control methods include botanical insecticides based on the leaf extracts of *Toona sinensis* L., *Tithonia diversifolia* (Hemsl.) A. Gray., and *Azadirachta indica* A. Juss [11]; phytohormones [12]; and various essential oils or their components [13,14]. Among the biological methods of control, the release of *Coccinella septempunctata* L. into the biotope [15] and the treatment of plants with the blastospores of *Verticillium lecanii* and *Beauveria bassiana* [16,17] have gained popularity. However, both chemical and biological methods have significant drawbacks: the first one negatively affects all components of the natural environment and contributes to the development of resistance [18], and the latter is often insufficiently effective, time-consuming, and expensive.

Considering the critical need for farming practices aimed at maximizing the yield of crops, pesticides from the following groups have been used successfully to chemically manage aphids worldwide: organophosphates, pyrethroids, neonicotinoids, and carbamates. 

Unfortunately, due to the development of resistance, the relative ineffectiveness of organophosphates [19], pyrethroids [20], neonicotinoids [21], and carbamates [22] against aphids is often detected.

Recently, our biotechnology based on antisense oligonucleotides, which appeared in 2008 [23], has gained popularity and distribution for crop protection. For example, there have been developments using a similar protection approach based on modified antisense oligonucleotides, called the 2′-deoxy-2′-fluoro-D-arabinonucleic acid (FANA) approach [24,25].

## 2. Materials and Methods

### 2.1. Origin of Material

The material for the research work was chrysanthemum seedlings infested with *M. sanborni*, obtained from the Nikita Botanical Garden (Yalta, Crimea). The experiment was carried out under laboratory conditions on isolated chrysanthemum plants at a temperature of 23 °C. The population density of the aphids on the chrysanthemums at the beginning of the experiment was 3.44 ± 1.08 individuals/cm^2^. The mortality was recorded daily.

### 2.2. Sequence Development

The design of Macsan-11 was carried out on the basis of the sequence (GeneBank: AB369150.1) using DNAinsector 1.0 software (www.dnainsector.com, accessed on 1 November 2022). Primers were selected using Primer Blast.

### 2.3. DNA Synthesis

DNA oligonucleotides and PCR primers (Table 1) were synthesized using the automatic DNA synthesizer ASM-800 (BIOSSET, Novosibirsk, Russia) with the standard phosphoramidite method on a universal solid carrier, UniLinker 500 Å (ChemGenes, Wilmington, MA, USA). The removal of oligonucleotides from the solid-phase carrier and the removal of protective groups were carried out overnight at 55 °C using a concentrated ammonia solution. After that, the solution was filtered and evaporated in a vacuum on a rotary evaporator (Heidolph, Schwabach, Germany). The resulting solid substance was dissolved in deionized Milli-Q water (Millipore, Molsheim, France) to the required concentration using a NanoDrop Lite spectrophotometer (Thermo Fisher Scientific, Waltham, MA, USA).

The structural accuracy of the synthesized DNA oligonucleotides was determined using a BactoSCREEN analyzer and a MALDI-TOF mass spectrometer (Lytech, Moscow, Russia). The ratio of mass (m) to charge (z) of the oligonucleotides was measured as positive ions with 3-hydroxypicolinic acid as the matrix on a LaserToFLT2 Plus device (UK) in a ratio of 2:1. The theoretical ratio, m/z, was calculated using the ChemDraw 18.0 program.

### 2.4. Sample Preparation and PCR

RNA isolation and cDNA synthesis were carried out in accordance with the manufacturers’ protocols using the ExtractRNA reagent (Eurogen, Moscow, Russia) and an RT-PCR kit (Syntol, Moscow, Russia) accordingly. RNA extraction was carried out in triplicate. Bands of RNA samples identical in size and number were obtained, which indicated the reproducibility of the RNA isolation from insects. For the cDNA synthesis, 10 µL of RNA was taken at a concentration of 20 ng/µL. Real-time PCR was performed to assess the expression of the target gene using 2.5×Reaction Mix for RT-PCR in the presence of SYBR Green I (Syntol, Moscow, Russia) in accordance with the manufacturer’s protocol on a real-time cycler LightCycler^®^ 96 (Roche, Rotkreuz, Switzerland), with the addition of an undiluted matrix.

The primers were annealed at a temperature of 53 °C (Agex_F–Agex_R) or at 62 °C (Agex_F–Agoss_R). The optimal temperature for each pair of primers was determined using gradient PCR. To evaluate the expression of target genes, the method described by the authors of [26] was used (Example 3).

### 2.5. Nuclease Activity

The nuclease activity was evaluated according to the following technique. The homogenate was prepared by grinding of 100 mg of insects in 1 mL of highly purified Milli-Q water and centrifuging for 5 min at 13,000 rpm. The homogenate was first incubated at 25 °C in a solid-state thermostat for 30, 45, 60, or 180 min, and then for 60 min at 90 °C for protein denaturation. After that, 9 µL of the homogenate was mixed with 3 µL of 4x Gel Loading Dye, Blue (Eurogen, Moscow, Russia) and applied to a 1.8% agarose gel. Electrophoretic separation was carried out using a voltage of 10 V/cm in a tris-borate buffer (1XTBE) for 40 min in a BlueMarine electrophoresis chamber (SERVA Electrophoresis GmbH, Heidelberg, Germany) with a Mini-300 power supply (Major Science, Taoyuan City, Taiwan). The nucleic acids in the agarose gel were visualized using a transilluminator (VilberLourmat, Marne-la-Vallée, France) and a 100 bp+ DNA marker (Evrogen, Moscow, Russia).

### 2.6. Statistics

For the statistical analyses, the mean and standard error of the mean (SE) were calculated using Student’s *t*-test; *p* < 0.01 was considered significant (Microsoft Excel software, Redmond, Washington, DC, USA).

### 2.7. Photos

The photos were taken using a stereoscopic microscope, the Saike Digital SK2100HDMI-T2H5 (Saike Digital Develop Tech, Shenzhen, China).

### 2.8. Sequencing

To confirm the complementarity of *M. sanborni* ITS2 to Macsan-11, a PCR product with a theoretical length of 399 bp was sequenced using Agex_F and Agoss_R primers.

## 3. Results

### 3.1. Synthesis of Oligonucleotides

The structural accuracy of the synthesized oligonucleotides was determined using the MALDI-TOF method, which showed that all oligonucleotides corresponded to their structures. The resulting ratios differed from the theoretically calculated ones by not more than 10 units (Table 2).

### 3.2. Evaluation of the Insecticidal Effect of Macsan-11 with Perfect Complementarity to the Target 

This is the first evidence for the effect of using antisense oligonucleotides for which the target is an ITS of a polycistronic rRNA transcript (PrRT).

**Figure 2:**
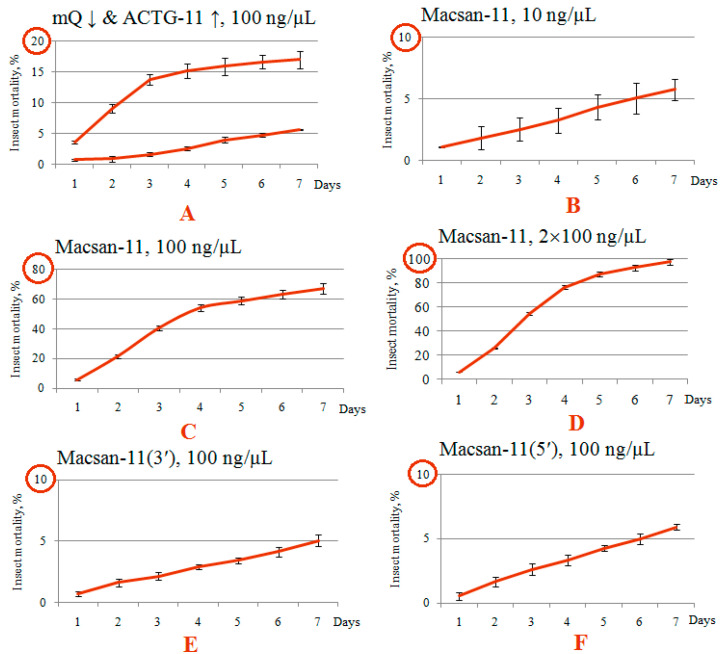
Dynamics of aphid mortality in groups of the experiment: (**A**–**F**)—different variants of the experiment.

The olinscide Macsan-11 targets the ITS2 of the PrRT between 5.8S and 28S of the chrysanthemum aphid. It showed a high efficiency, leading to a mortality rate of 67.15 ± 3.32% for insect pest individuals (χ2 = 448.8, *p* < 0.001, N = 1100, df = 1) by the 7th day of the experiment after a single treatment with Macsan-11 at a concentration of 100 ng/μL, and 97.38 ± 2.49% (χ2 = 360.9, *p* < 0.001, N = 1100, df = 1) by the 7th day of the experiment after a double treatment with the same solution on a daily interval (Figure 2). The maximum mortality of insects was noted on the 3rd day after the beginning of the experiment, and was 18.73 ± 0.41% in the group with one treatment and 28.82 ± 1.02% in the group with two treatments. It should be noted that Macsan-11 at a concentration of 10 ng/µL did not have any insecticidal effect on chrysanthemum aphid mortality, resulting in a mortality rate of 5.79 ± 0.86% (χ2 = 0.01, *p* = 0.916, N = 825, df = 1). Thus, the LD50 for Macsan-11 lies between the concentrations of 10 and 100 ng/μL.

### 3.3. Pronounced Decrease in Insecticidal Effect for Olinscides without Perfect Complementarity to the Target

To confirm the high selectivity of antisense oligonucleotides, the insecticidal effect of two oligonucleotides similar to Macsan-11—Macsan-11(3′) and Macsan-11(5′)—were used. They differed from Macsan-11 by substitutions of nitrogenous bases at the 3′- and 5′-ends (Figure 2E,F). In the Macsan-11(5′) oligonucleotide, adenine was replaced with thymine, and in the Macsan-11(3′) oligonucleotide, thymine was replaced with adenine. They had no significant effect on aphid mortality in comparison with the control. After 7 days, the mortality reached 5.07 ± 0.47% (*p* > 0.05) and 5.92 ± 0.25% (*p* > 0.05) for Macsan-11(3′) and Macsan-11(5′), respectively. The obtained mortality rates corresponded to the mortality rate in the control group.

A moderate mortality rate (17 ± 1.38%) for chrysanthemum aphid control after treatment with a fragment of ACTG-11 (Figure 2A) was shown and can be explained by the fact that this oligonucleotide is complementary to fragments of the genes of the gamma-aminobutyric acid type B receptor subunit, protein kinase C-binding protein NELL1, GATA protein of the zinc finger domain 10, ADP ribosylation factor, and other genome sequences of representatives of the Aphididae family (according to GenBank). At the same time, these data show that the ITS2 of the PrRT is a more suitable target for the action of olinscides.

In addition, to evaluate the possible negative effects of the Macsan-11 insecticide on the plant, we measured the pH of the leaves (Table 3). No significant difference was found, confirming the environmental safety of the Macsan-11 insecticide (*p* > 0.05).

### 3.4. Assessment of Nuclease Activity

**Figure 3:**
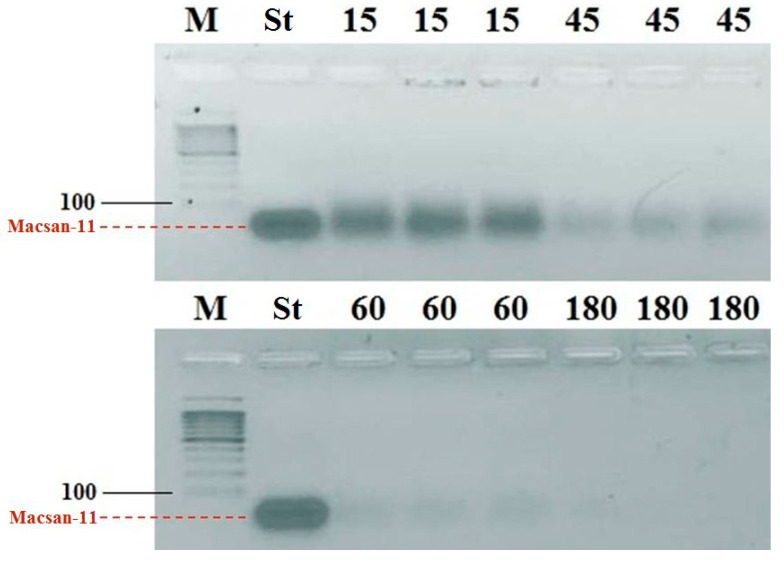
Nuclease activity of chrysanthemum aphid homogenates. M—marker of DNA lengths of 100 bp+; St—standard of Macsan-11, 170 ng/μL; 15, 45, 60, 180 —mixtures (V:V—1:1) of homogenate and Macsan-11 (340 ng/μL) incubated at 25 °C for 15, 45, 60, or 180 min, respectively. The experiment was repeated in triplicate.

An analysis of the nuclease activity of aphid tissue homogenates (Figure 3) showed that, within the short time period of less than three hours, cellular DNases completely degraded the DNA oligonucleotides that entered the body.

### 3.5. Evaluation of Concentration of Target RNA

**Figure 4:**
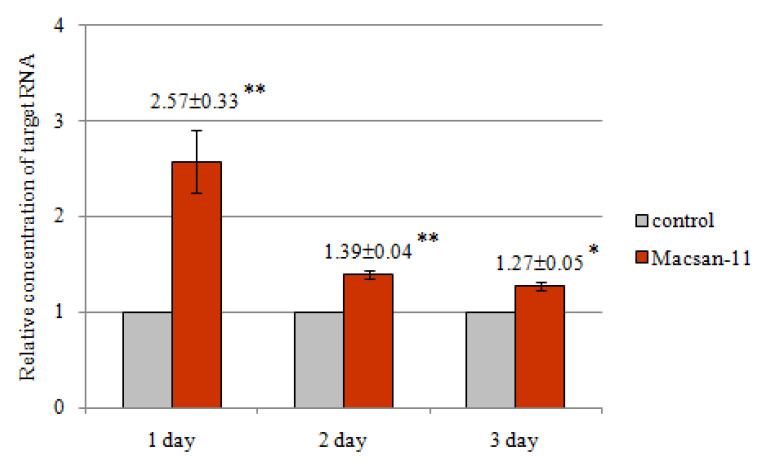
The concentration of the target RNA ITS2 of PrRT after Macsan-11 treatment at a concentration of 100 ng/μL; * *p* < 0.05; ** *p* < 0.01; control was taken as 1.

It was found that the concentration of the target RNA 3 days after the treatment was higher than in the control (Figure 4). On the 1st day, the concentration was 2.57 ± 0.33 times higher in comparison with the control. Then, this indicator gradually decreased—it was 1.39 ± 0.04 times higher in comparison with the control on the 2nd day and 1.27 ± 0.05 times higher in comparison with the control on the 3rd day. Thus, the imbalance in the concentration of the target RNA gradually leveled, but was enough to cause significant mortality in the group treated with Macsan-11.

### 3.6. Sequencing of the Target Fragment of ITS2 in Chrysanthemum Aphids

**Figure 5:**
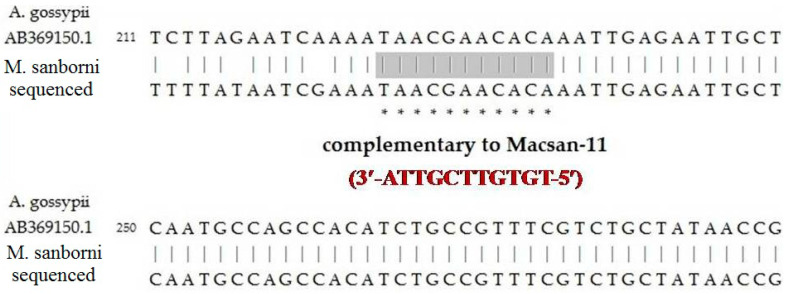
Alignment of the sequenced ITS2 fragment of *M. sanborni* with the reference sequence of *A. gossypii* (GenBank: AB369150.1) and Macsan-11; gray part and * indicate region of sequences complementary to Macsan-11.

As a result of DNA sequencing, a DNA fragment was obtained and submitted to GenBank (GenBank: OR046519), part of which is shown in Figure 5. The perfect complementarity of the target gene to the developed oligonucleotide insecticide Macsan-11 was confirmed.

## 4. Discussion

In earlier studies [1,27], insect mortality was regulated only with the help of olinscides complementary to sites of mature rRNA. In this case, we saw something striking: since the ITS2 is immediately degraded after rRNA maturation, it is obvious that antisense oligonucleotides mainly affect the ITS2 at the stage of the PrRT. Nevertheless, the oligonucleotide Macsan-11, which triggers the ITS2, demonstrated its high potential for plant protection, and this technology has been presented as one of the most promising biotechnologies that enables insect pest control without harm to the environment.

It is worth noting that viviparous aphid females have a greater viability than the larval stages. The females continued to reproduce parthenogenetically, thereby filling the population with larvae that were not in contact with Macsan-11. Obviously, the insecticidal load was too small for newborn nymphs. The introduction of the group with a double treatment allowed a larger number of individuals to be affected. The analysis of the mortality curves showed that, after the use of Macsan-11, the formation of an S-shaped mortality curve was observed and its angle of inclination was close to 45° on the 4th day. With the double treatment, we observed a more pronounced exponential increase and the same level of mortality was reached on the 3rd day (Figure 2C,D).

A visual illustration of the work of DNases using gel electrophoresis showed that, in fact, the action of the Macsan-11 oligonucleotide as a blocker of the ITS2 of the target RNA is limited in time by the action of cellular enzymes from the aphid tissue homogenate. They degrade it, but this time is enough to provoke a long-term effect and disable the work of the protein biosynthesis of the cell. It is also possible that in an intact cell, DNA oligonucleotides persist for a longer time and may have a prolonged insecticidal effect. The fine details of this mechanism are being investigated; however, the eco-friendliness and fast biodegradability of olinscides make it possible to actively introduce this class of insecticides to regulate the number of insect pests.

The results found for oligonucleotide insecticides on *Coccus hesperidum* obtained by the evaluation of gene expression are partly consistent with our results for Coccus-11 [23], and demonstrate an increase in the expression on the 2nd day after exposure to the olinscide, with its subsequent gradual decrease. Macsan-11 up-regulated the expression of the target gene, which probably indicates molecular hypercompensation in response to the stress factor due to the fact that vital genes involved in protein biosynthesis and oxidative phosphorylation were affected. Apparently, insect tissues try to replace dead cells, which ultimately leads to an increase in the expression of genes important for the growth of the organism, an imbalance in the proportion of transcripts, cell depletion, and insect death.

## 5. Conclusions

The effect of the oligonucleotide insecticide Macsan-11 complementary to the ITS2 of the PrRT on the expression of the target gene and the subsequent death of the targeted insect pest has been shown for the first time. This study generally showed the sensitivity of cells to antisense oligonucleotides, and the greatest effect occurred in the case of perfect complementarity of the antisense oligonucleotide to the target gene.

During the study, the pronounced insecticidal effect of the olinscide Macsan-11 was shown. With the help of the Macsan-11(3′) and Macsan-11(5′) oligonucleotides, a high selectivity was demonstrated, as determined by the absence of an insecticidal effect when replacing even one nitrogenous base out of eleven, which does not allow the olinscide to work effectively. The obtained results indicate the safety of Macsan-11 for non-target organisms and, at the same time, make it possible to create universal oligonucleotide insecticides for closely related insect pest species based on the insects’ genomes. It has been shown that aphid DNases degrade Macsan-11 in less than 3 h, which demonstrates a high level of biodegradability for the olinscide. The established jumps in the expression of RNA targets show how the aphid organism tries to hypercompensate for the effect of the antisense oligonucleotides. The fine details of this mechanism are being investigated, but it is obvious that the protein biosynthesis reacts rapidly to the penetration of the oligonucleotide insecticides and is very sensitive to them. The importance of the relevant dose (100 ng/μL) and the double treatment with the olinscide Macsan-11 on the effective control of chrysanthemum aphids has been demonstrated.

The development and registration of olinscides against insect pests from the suborder *Sternorrhyncha* is a direction of plant protection. With an increase in the number of elucidated sequences of insect genomes in GenBank, the development of this class of insecticides will gain even greater reliability and a wide distribution, since they rely not only on a single sequence of an insect pest, but also on data collected by researchers from different parts of the world.

## Figures and Tables

**Table 1:** Characteristics of DNA oligonucleotides used in the experiment with *Macrosiphoniella sanborni*.

Sequence		Target
**Macsan-11**	5′-TGTGTTCGTTA-3′	ITS2 ^1^ of polycistronic rRNA transcript
Macsan-11(3′)	5′-TGTGTTCGTTT-3′	ITS2 (changed A to T at 3′ end of Macsan-11)
Macsan-11(5′)	5′-AGTGTTCGTTA-3′	ITS2 (changed T to A at 5′ end of Macsan-11)
ACTG-11	5′-ACTGACTGACT-3′	Identification of the insecticidal effect of a random DNA oligonucleotide
Agex_F	5′-TGCAAGTGCGCTTCCACTTA-3′	Forward primer for sequencing and concentration evaluation/ITS2
Agex_R	5′-TAGCAGACGAAACGGCAGAT-3′	Reverse primer for concentration evaluation/ITS2
Agoss_R	5′-ACGGGGACATCGTGATTTTG-3′	Reverse primer for sequencing/ITS2

^1^ ITS2—internal transcribed spacer 2.

**Table 2:** Results of the analysis of synthesized oligonucleotides by the MALDI-TOF method.

	Name	Theoretical m/z Ratio	Resulting m/z Ratio
Olinscides	Macsan-11	3343.57	3345.93
	Macsan-11(3′)	3343.57	3339.26
	Macsan-11(5′)	3361.60	3367.41
	ACTG-11	3315.60	3321.04
Primers	Agex_F	6067.05	6062.50
	Agex_R	6183.10	6178.09
	Agoss_R	6187.06	6192.74

**Table 3:** The results for measuring the pH of *C. morifolium* leaves.

Day	Control (mQ Water)	Macsan-11
1st	5.20 ± 0.01	5.18 ± 0.03
7th	5.21 ± 0.05	5.20 ± 0.02

## Data Availability

The parts of the datasets generated and/or analyzed during the current study are available in the Mendeley Data repository, https://data.mendeley.com/datasets/kxs784r2gs/1, accessed on 1 November 2022.

## References

[1] Oberemok V., Laikova K., Useinov R., Gal’chinsky N., Novikov I., Gorlov M. (2020). High Mortality of Sap-sucking Insects One Week After Topical Application of DNA Insecticides. Vitr. Cell. Dev. Biol.–Anim..

[2] Blackman R.L., Eastop V.F. (1984). Aphids on the World’s Crops: An Identification and Information Guide.

[3] Chan C.K., Forbes A.R., Raworth D.A. (1991). Aphid-transmitted viruses and their vectors of the world. Agric. Canada Res. Branch Tech. Bull..

[4] Chatzivassiliou E.K., Papapanagiotou A.P., Mpenardis P.D., Perdikis D.C., Menexes G. (2016). Transmission of Moroccan watermelon mosaic virus (MWMV) by aphids in Greece. Plant Dis..

[5] Volesky N., Schrumm Z.R. (2021). High Tunnel Pest Management-Aphids. Utah Pest Fact Sheet. Utta State Univ..

[6] Zhong J., Wang Y., Lu Y., Ma X., Zhang Q., Wang X., Zhang Q., Sun M. (2022). Identification and Expression Analysis of Chemosensory Genes in the Antennal Transcriptome of Chrysanthemum Aphid *Macrosiphoniella sanborni*. Insects.

[7] Zhang W., Gao T., Li P., Tian C., Song A., Jiang J., Guan Z., Fang W., Chen F., Chen S. (2020). Chrysanthemum *CmWRKY53* negatively regulates the resistance of chrysanthemum to the aphid *Macrosiphoniella sanborni*. Hortic. Res..

[8] Rene E.R., Bui X.T., Ngo H.H., Nghiem L.D., Guo W. (2021). Green technologies for sustainable environment: An introduction. Environ. Sci. Pollut. Res. Int..

[9] Dhakal R., Ghimire R., Sapkota M., Thapa S., Bhatta A.K., Regmi R. (2019). Bioefficacy of different insecticides on cowpea aphid (*Aphis craccivora* Koch). Int. J. Entomol. Res..

[10] Naveena J.B. (2019). Sharanabasappa Effect of different insecticides against aphids, *Aphis gossypii* and whiteflies, *Bemisia tabaci*. J. Pharmacog. Phytochem..

[11] Rahardjo I.B., Hutapea D., Marwoto B., Budiarto K. (2021). Effects of Several Botanical Insecticides Applied in Different Periods to Control Aphids (*Macrosiphoniella sanborni* Gillette) on Chrysanthemum. AGRIVITAJ Agricult. Sci..

[12] Fan J., Zhang X.Y., Sun X.Z., Xu B.Y. (2020). Effect of methyl jasmonate on aphid resistance of chrysanthemum. Ying Yong Sheng Tai XueBao.

[13] Zhang X.Y., Shen J., Zhou Y., Wei Z.P., Gao J.M. (2017). Insecticidal Constituents from *Buddlej aalbiflora* Hemsl. Nat. Prod. Res..

[14] Ikbal C., Pavela R. (2019). Essential oils as active ingredients of botanical insecticides against aphids. J. Pest. Sci..

[15] Emam A.K. (2016). Biological Control of the Chrysanthemum Aphid, Macrosiphoniellasanborni (Gillete) by Release *Coccinella septempunctata* L. on Chrysanthemum Plants. Pathol..

[16] Helyer N., Gill G., Bywater A. (1992). Elevated humidities for control of chrysanthemum pests with *Verticillium lecanii*. Pesticide Sci..

[17] Sudan Y.E., Yuhui D., Feng M.G. (2005). Time and concentration dependent interactions of *Beauveria bassiana* with sublethal rates of imidacloprid against the aphid pests *Macrosiphoniella sanborni* and *Myzus persicae*. Ann. Appl. Bio..

[18] Schulz R., Bub S., Petschick L.L., Stehle S., Wolfram J. (2021). Applied pesticide toxicity shifts toward plants and invertebrates, even in GM crops. Science.

[19] Kaleem Ullah R.M., Gao F., Sikandar A., Wu H. (2023). Insights into the Effects of Insecticides on Aphids (Hemiptera: Aphididae): Resistance Mechanisms and Molecular Basis. Int. J. Mol. Sci..

[20] Sekamatte M.B., Ogenga-Latigo M. Efficacy and Impact of Some Insecticides Used to Control Aphids, *Aphis gossypii* Glover (Homopters: Aphididae) on Cotton in Uganda, on Predators. Proceedings of the World Cotton Research Conference-2.

[21] Sial M.U., Mehmood K., Saeed S., Husain M., Rasool K.G., Aldawood A.S. (2022). Neonicotinoid’s resistance monitoring, diagnostic mechanisms and cytochrome P450 expression in green peach aphid [*Myzus persicae* (Sulzer) (*Hemiptera: Aphididae*)]. PLoS ONE.

[22] Umina P.A., Edwards O., Carson P., Van Rooyen A., Anderson A. (2014). High levels of resistance to carbamate and pyrethroid chemicals widespread in Australian *Myzus persicae* (*Hemiptera: Aphididae*) populations. J. Econ. Entomol..

[23] Oberemok V.V. (2008). Ukrainian Patent Application.

[24] Hunter W.B. (2021). Improving Suppression of Hemipteran Vectors and Bacterial Pathogens of Citrus and Solanaceous Plants: Advances in Antisense Oligonucleotides (FANA). Front. Agron..

[25] Sandoval-Mojica A.F. (2021). Antibacterial FANA oligonucleotides as a novel approach for managing the Huanglongbing pathosystem. Sci. Rep..

[26] Schmittgen T.D., Livak K.J. (2008). Analyzing real-time PCR data by the comparative C(T) method. Nat. Protoc..

[27] Oberemok V.V., Useinov R.Z., Skorokhod O.A., Gal’chinsky N.V., Novikov I.A., Makalish T.P., Yatskova E.V., Sharmagiy A.K., Golovkin I.O., Gninenko Y.I. (2022). Oligonucleotide Insecticides for Green Agriculture: Regulatory Role of Contact DNA in Plant-Insect Interactions. Int. J. Mol. Sci..

